# Classification and clinical significance of intracranial calcifications: a pictorial essay

**DOI:** 10.1590/0100-3984.2019.0094

**Published:** 2020

**Authors:** Marcelo dos Santos Guedes, Isadora Carvalho Queiroz, Cláudio Campi de Castro

**Affiliations:** 1 Hospital Alvorada, São Paulo, SP, Brazil.; 2 Instituto do Coração do Hospital das Clínicas da Faculdade de Medicina da Universidade de São Paulo (InCor/HC-FMUSP), São Paulo, SP, Brazil.

**Keywords:** Intracranial calcifications, Computed tomography, Magnetic resonance imaging, Calcificações intracranianas, Tomografia computadorizada, Ressonância magnética

## Abstract

Intracranial calcifications, which are common in the daily routine of radiologists, can have a physiological or pathological origin. Determining the cause of intracranial calcifications can represent a challenge. The anatomical location, distribution, dimensions and morphology of such calcifications are important findings, which, in conjunction with the clinical history and age group, can facilitate the differential diagnosis. The aim of this pictorial essay is to demonstrate the different types of intracranial calcifications and their origins. The images evaluated were those stored in picture archiving and communication systems. All of the cases included were studied by computed tomography, magnetic resonance imaging, or both. We identified, classified, and described 64 types of intracranial calcifications.

## Abstract (pt)

Calcificações intracranianas fazem parte da rotina do médico radiologista, as quais podem ter origem fisiológica ou patológica. A diferenciação entre as calcificações pode representar um desafio. Localização anatômica, distribuição, dimensões e morfologia das calcificações são importantes achados que, associados a história clínica e faixa etária, podem ajudar no diagnostico diferencial. O objetivo deste estudo é demonstrar os diferentes tipos de calcificações intracranianas e suas origens. Foram avaliadas as imagens armazenadas no sistema de comunicação e arquivamento de imagens das unidades. Todos os casos inclusos foram estudados por tomografia computadorizada e/ou ressonância magnética. Foram encontrados 64 tipos de calcificações intracranianas, que foram classificadas e descritas.

## INTRODUCTION

Intracranial calcifications, which are commonly seen in the daily routine of radiologists, can have a physiological or pathological origin^(1,2)^. Determining the cause of intracranial calcifications can represent a challenge, because calcifications that are categorized as physiological, depending on patient age, can have other causes. The anatomical location, distribution, dimensions, and morphology of such calcifications are important findings that, in conjunction with the clinical history and age group, can facilitate the differential diagnosis^(2)^.

The technological advances of recent decades, especially in imaging methods, have promoted an improvement in the sensitivity and specificity of their assessment, which facilitates the diagnosis and reduces the possibility of errors of conduct^(3)^.

The objective of this pictorial essay is to demonstrate and classify the different types of intracranial calcifications. That knowledge will allow physiological findings to be differentiated from pathological findings.

We reviewed multidetector computed tomography (CT) and magnetic resonance imaging (MRI) scans of the skull acquired between January 2012 and February 2019. The images evaluated were those stored in the picture archiving and communication systems of the participating facilities. Cases were included only if the scans showed calcifications and the diagnosis had been established on the basis of pathological, biochemical, or radiological findings, when pathognomonic. The cases were then divided into groups, based on imaging characteristics, clinical data, and age group, according to their etiology and didactic purpose. A total of 64 types of physiological or pathological intracranial calcifications were detected.

## PHYSIOLOGICAL AND AGE-RELATED CALCIFICATIONS

**Figure 1:**
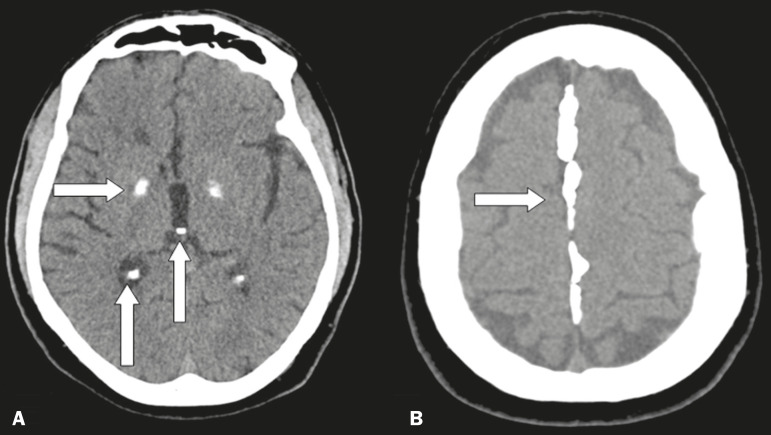
Unenhanced axial cranial CT scans, with soft-tissue window settings, showing physiological calcifications in the globus pallidus, choroid plexus, and pineal gland/habenulae (arrows in **A**), as well as in the cerebral sickle (arrow in **B**).

Physiological and age-related calcifications are typical findings in adult and elderly patients. They can be found in the choroid plexus, pineal gland, habenulae, globus pallidus, tentorium, petroclinoid ligament, cerebral sickle, and the dura mater (Figure 1). However, they are especially prevalent in the pineal gland, where they are typically seen in individuals ≥ 10 years of age, and in the globus pallidus, where they are typically seen in individuals ≥ 40 years of age. These types of calcifications can take on a variety of radiological aspects, appearing as punctiform, confluent, nodular, coarse, or sometimes extensive images^(1)^.

## PATHOLOGICAL CALCIFICATIONS

### Infections

**Figure 2:**
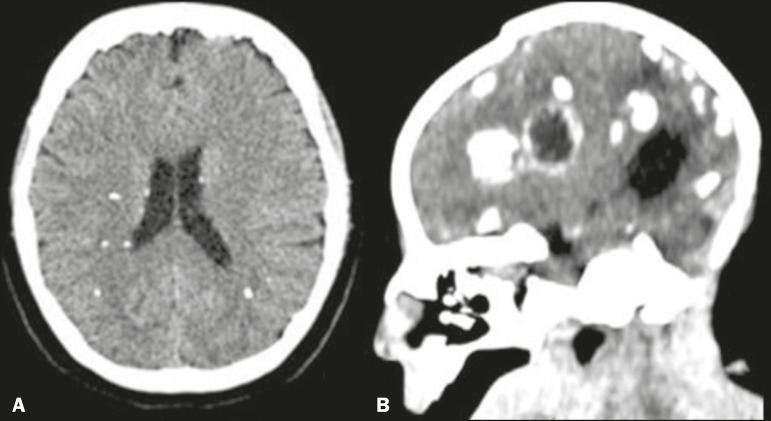
Unenhanced axial and sagittal cranial CT scans (**A** and **B**, respectively), with soft-tissue window settings, showing periventricular and particularly subcortical calcifications, in a case of neurocysticercosis (**A**), as well as periventricular and cortical (sometimes rope-like), calcifications in a child with microcephaly with an apparently “collapsed” skull, in a case of congenital Zika virus infection (**B**).

Certain types of infections are commonly associated with calcifications, one of the most characteristic representatives of this group being the toxoplasmosis, rubella, cytomegalovirus, and herpes complex, within which toxoplasmosis and cytomegalovirus predominate. However, there are a number of other infectious causes of calcifications, such as neurocysticercosis, syphilis, varicella zoster, human T-lymphotropic virus I, tuberculosis, HIV, and the Zika virus, we should emphasize the high incidence of neurocysticercosis in Brazil. Calcifications in patients with neurocysticercosis are usually of the nodular type (Figure 2), and their topography is quite relevant to making the differential diagnosis^(4)^.

### Vascular

**Figure 3:**
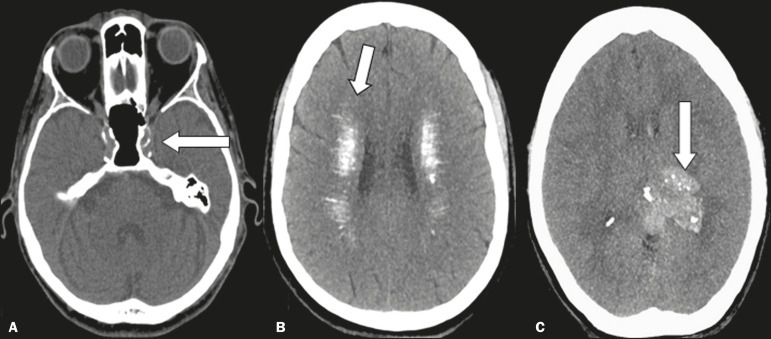
Unenhanced axial cranial CT scans, with soft-tissue window settings, showing calcifications on the walls of blood vessels (arrow) in a case of atherosclerosis (**A**), multiple punctiform and elongated calcifications, with extensive areas of confluence in both brain hemispheres (arrows) in a case of lupus (**B**), and punctiform vascular calcifications, some nodular (arrow), in a case of arteriovenous malformation (**C**).

There are a large number of vascular diseases that can present calcifications with various aspects, ranging from punctiform, peripheral calcifications, with a gyral morphology, to extensive calcifications (Figure 3), which can be related to atherosclerosis, aneurysm, hematoma, stroke, developmental venous anomalies, cavernoma, arteriovenous malformations, telangiectasia, lupus, calcified embolism, and hypoxic-ischemic events, atherosclerosis being the most prevalent^(2,5)^.

### Metabolic and endocrine disorders

**Figure 4:**
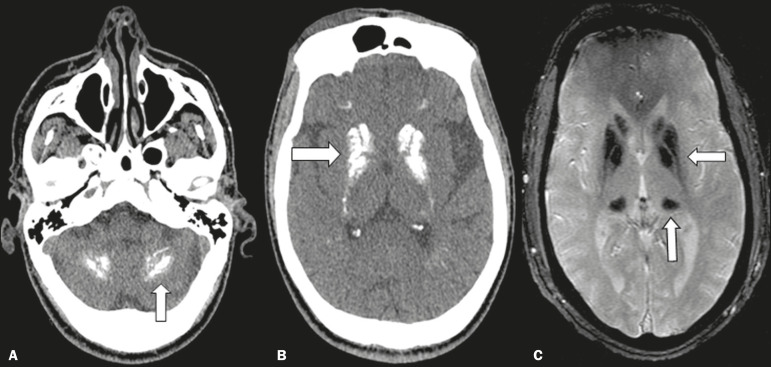
Unenhanced axial cranial CT scans, with soft-tissue window settings (**A,B**), and axial T2*-weighted gradient-recalled echo MRI sequence of the skull (**C**), showing confluent calcifications in the region of dentate nuclei (arrow) in a case of pseudo-hypoparathyroidism (**A**), confluent calcifications in the area of basal ganglia (arrow) in a case of Fahr’s disease (**B**), and areas of markedly hypointense signals, corresponding to calcifications in the region of the basal ganglia and pulvinar of the thalamus (arrows), in a case of Fabry disease (**C**).

The metabolic and endocrine disorders group includes three diseases that have overlapping radiological signs and are therefore difficult to distinguish visually: Fahr’s disease; hypoparathyroidism and its variants; and Fabry disease (Figure 4), all of which are characterized by bilateral, symmetric deposition of calcium in the dentate core, basal ganglia, thalamo, and subcortical white matter^(6)^.

### Neurocutaneous syndromes

**Figure 5:**
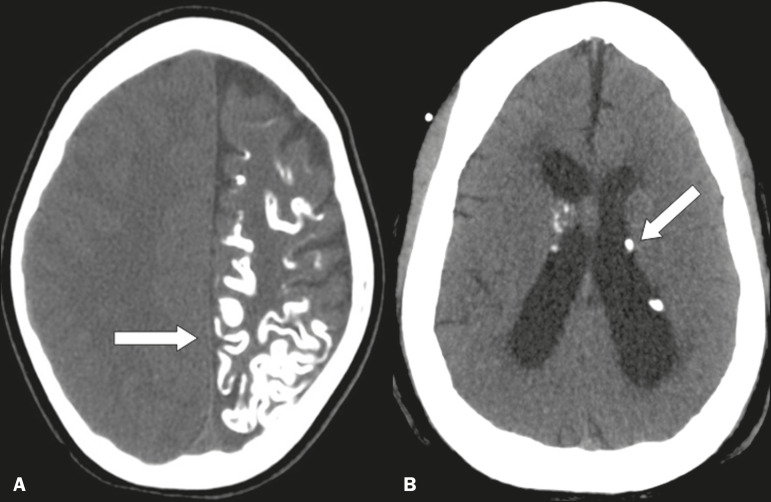
Unenhanced axial cranial CT scans, with soft-tissue window settings, showing gyral calcifications (arrow) with parenchymal atrophy in a case of Sturge-Weber syndrome (**A**), as well as small intraventricular, periventricular, and subependymal calcifications (arrows) in a case of tuberous sclerosis (**B**).

There are a number of diseases that share signs of neurological and cutaneous involvement, some of them presenting with distinct calcifications that allow the definitive diagnosis of diseases such as Sturge-Weber syndrome, tuberous sclerosis, neurofibromatosis (type I and II), and Gorlin-Goltz syndrome (Figure 5), the last one is characterized by rough calcifications in the cerebral sickle, accompanied by odontogenic cutaneous lesions, in young patients^(2,7)^.

### Neurometabolic and rare syndromes

**Figure 6:**
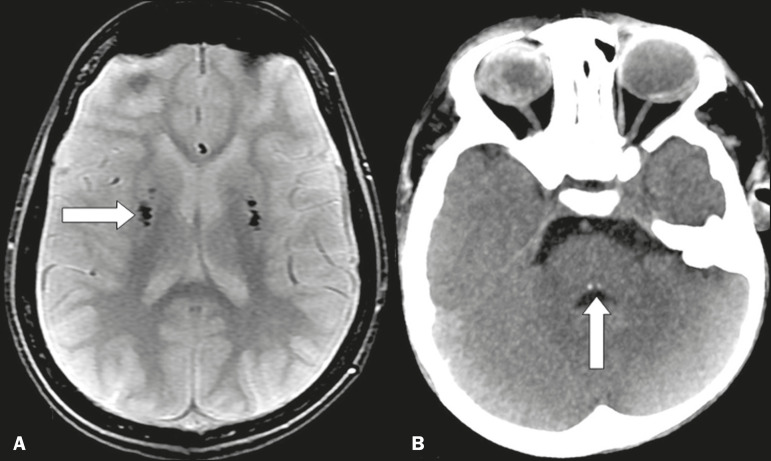
**A:** Axial T2*-weighted gradient-recalled echo MRI sequence of the skull, showing areas of markedly hypointense signals, corresponding to calcifications in the area of the lentiformis nuclei (arrow) in a case of Cockayne syndrome. **B:** Unenhanced axial cranial CT scan, with soft-tissue window settings, showing small calcifications in the posterior aspect of the brainstem, in the region of the abducens nuclei (arrow), in a case of Moebius syndrome.

Neurometabolic syndromes rarely present with calcifications, notable exceptions being Cockayne syndrome, in where there are globus pallidus calcifications, and leukoencephalopathy of Duane, in where there are periventricular cysts and calcifications (Figure 6). We can also mention calcifications associated with other uncommon syndromes, such as Nasu-Hakola disease, Urbach-Wiethe disease, and Moebius syndrome, the last being a congenital malformation in which punctiform calcifications can be seen in the posterior brainstem, at the site of the abducens nucleus^(2,8)^. Also noteworthy for their clinical relevance are calcifications of the mesial temporal/hippocampus region, particularly in the context of epilepsy, which can be physiological (e.g., calcifications in the choroid plexus) or pathological (e.g., calcifications stemming from infectious or neoplastic sequelae).

### Non-neoplastic expansive lesions

**Figure 7:**
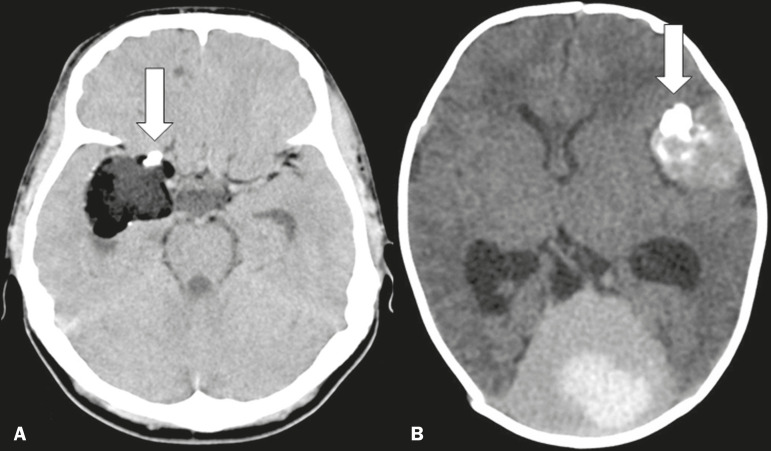
Unenhanced axial cranial CT scans, with soft-tissue window settings, showing lesion with fat attenuation and calcified nodules (arrow) in its anterior aspect, in a case of dermoid cyst (**A**), as well as expansile, hemorrhagic, formations within the meninges, some with odontogenic calcifications (arrow), in a case of hemorrhagic hamartomatous meningioangiomatosis (**B**).

There are a number of lesions that, although not neoplastic, have an expansive character that can occasionally show calcifications, predominantly nodular but also rough or even odontogenic. Such lesions include dermoid cysts, epidermoid cysts, and, in some presentations, colloid cysts of the third ventricle, as well as meningioangiomatosis, which is a rare hamartomatous lesion^(2,9)^, as depicted in Figure 7.

### Neoplastic lesions

**Figure 8:**
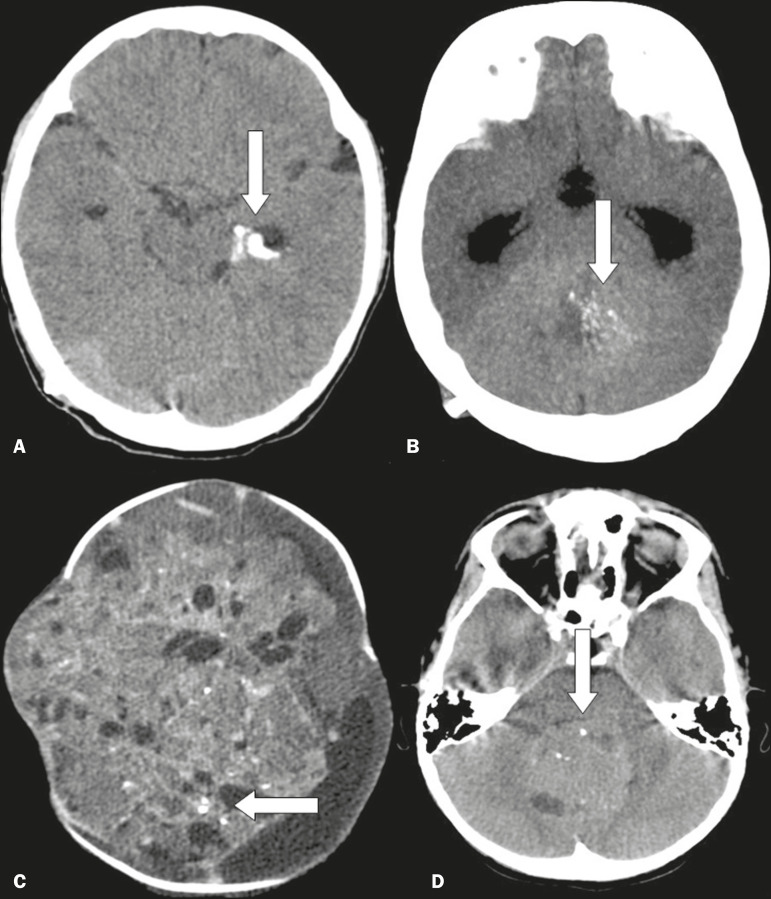
Unenhanced axial cranial CT scans, with soft-tissue window settings, showing coarse, peripheral calcifications (arrow), together with a temporal cystic lesion, in a case of ganglioglioma (**A**), multiple, small confluent calcifications (arrow) in an expansive lesion in the posterior fossa, in a case of ependymoma (**B**), small, sparse calcifications (arrows) in a voluminous expansive supratentorial lesion in a child with a pineal gland tumor (**C**), and small sparse calcifications (arrow) in an expansive lesion in the posterior fossa in a case of medulloblastoma (**D**).

A number of neoplastic lesions can present with various types of calcifications^(2,10)^, such as the punctiform, nodular, and coarse types, and the calcification can be extensive. Such lesions include the following (Figure 8): meningioma; craniopharyngioma; pilocytic astrocytoma; oligodendroglioma; ganglioglioma; ependymoma; subependymal giant cell astrocytoma; choroid plexus papilloma/carcinoma; teratoma; medulloblastoma and other embryonal tumors; pineocytoma; pineoblastoma; chordoma; and metastases.

## CONCLUSION

Intracranial calcifications can manifest in a variety of situations, and it is essential that the radiologist know how to identify and classify them as accurately as possible. To that end, radiologists should consider the imaging characteristics of these calcifications, the age of the patient, and the clinical aspects. We can thus avoid practical errors and facilitate the differential diagnosis.

## References

[1] Sedghizadeh PP, Nguyen M, Enciso R (2012). Intracranial physiological calcifications evaluated with cone beam CT. Dentomaxilofac Radiol.

[2] Chattopadhyay A, Coates J, Craven I (2018). Intracranial calcifications - a pictorial review.

[3] Bradley WG Jr, Bydder GM (1997). Advanced MR imaging techniques.

[4] Fink KR, Thapa MM, Ishak GE (2010). Neuroimaging of pediatric central nervous system cytomegalovirus infection. Radiographics.

[5] Geibprasert S, Pongpech S, Jiarakongmun P (2010). Radiologic assessment of brain arteriovenous malformations: what clinicians need to know. Radiographics.

[6] Hegde AN, Mohan S, Lath N (2011). Differential diagnosis for bilateral abnormalities of the basal ganglia and thalamus. Radiographics.

[7] Umeoka S, Koyama T, Miki Y (2008). Pictorial review of tuberous sclerosis in various organs. Radiographics.

[8] van der Knaap MS, Valk J (2005). Magnetic resonance of myelination and myelin disorders.

[9] Osborn AG, Preece MT (2006). Intracranial cysts: radiologic-pathologic correlation and imaging approach. Radiology.

[10] Greenberg HS, Chandler WF, Sandler HM (2009). Brain tumors.

